# Geographies of the global co-editor network in oncology

**DOI:** 10.1371/journal.pone.0265652

**Published:** 2022-03-17

**Authors:** György Csomós, Balázs Lengyel

**Affiliations:** 1 Department of Civil Engineering, University of Debrecen, Debrecen, Hungary; 2 Agglomeration and Social Networks Lendület Research Group, Centre for Economic- and Regional Studies, Budapest, Hungary; 3 Centre for Advanced Studies, Corvinus University of Budapest, Budapest, Hungary; University of Siena, Italy, ITALY

## Abstract

The co-editor networks of academic journals are generally examined at the journal level. This paper investigates the geographies of the global co-editor network in oncology through the lens of cities. After using different network methods to analyze the global co-editor network, we found that the network can be characterized by a core-periphery structure. The dense core is occupied by many highly interconnected cities, whereas the periphery contains many cities maintaining loose connections with the core cities. The core shows an asymmetric dual sub-core structure. The greater sub-core is constituted by Northern American cities with New York, Washington DC, Boston, Houston, and Los Angeles in the center, whereas the smaller sub-core is formed by Asian cities and centered on Tokyo, Seoul, Osaka, Beijing, and Shanghai. The European core cities do not form a well-outlined sub-core but produce a ringlike shape around the Northern American core. This structure of the co-editor network is a consequence of the prestige effect still characterizing global science. Many European and Chinese journals tend to employ Northern American editors (US-based editors in the first place) to help increase the reputation of the journal. However, US-based journals are more interested in recruiting American editors from the top-ranked national cancer centers and universities rather than outside of the country.

## 1. Introduction

Science in our age is characterized by such fundamental attributes as collaboration, internationalization, and multipolarity. It has been widely demonstrated that the ratio of co- and multi-authored papers has been growing rapidly (we have recently witnessed the publication of a paper produced by more than 5,000 authors) [1,2]. In the past few decades, an increasing number of countries (including post-socialist, newly industrialized, and many developing countries) have entered the arena of global science. [3]. Besides experiencing a robust increase in international scientific collaborations [4,5], global science has become even more multipolar [6]. China has risen as a scientific power, the United States (US) has still managed to maintain its dominance, whereas the European Union (EU) strives to preserve its position in this currently tripolar system [7–10]; that is, the global science can be characterized not only by collaboration but also fierce competition between the main actors.

The above attributes of science (i.e., collaboration, internationalization, and multipolarity) are most often observed and evaluated through the lens of scientific publications. Collaboration is investigated based on co-authorship characteristics, the increasing number of countries that participate in the production of publications reflects on the internationalization of science, and the multipolarity of the system is generally expressed by comparing the number of publications produced or citations received by different countries. Science production (e.g., the production of scientific publications) is, however, only one side of the coin, whereas less attention is given to the attributes of gatekeepers, who control the main communication channels of scientific information [11,12]. The gatekeepers are generally identified as the editorial board members and reviewers of scientific journals and have the power to filter scientific information by deciding whether a manuscript being submitted to a journal will be accepted (instantly or after modifications) or rejected.

The editorial board of a scientific journal can be understood as a network of professionals representing a particular discipline (or a couple of related disciplines) [13]. The network is generally hierarchical because the final decision is generally in the hands of the editor-in-chief(s) (except for some megajournals, such as PLoS ONE, where the decision-making power is placed down at the manuscript level) [14]. Naturally, a researcher can hold membership in more than one journal’s editorial board, making the two or more journals’ editorial boards interconnected. In some fields containing many journals, due to the high degree of overlap between the editorial boards, an extensive interlocking editorship network will be produced [15,16].

Recently, the investigation of the characteristics of interlocking editorship networks has gained widespread attention. In the field of financial economics, Andrikopoulos and Economou [17] detected a core−periphery structure of editors of 20 journals by employing a network analysis. Goyanes and de-Marcos [18] conducted a social network analysis to reveal the interlocking structure of 41 communication journals’ editorial boards. By mapping interlocking editorial board social networks in knowledge management and intellectual capital fields, Teixeira and Oliveira [19] explored which journals were the most influential. Zhang and Jiang [20] constructed a social network matrix of the editorial board members of 23 journals in the field of library and information science and then utilized a K-core analysis to separate the network structure and find the core subgroup. Ni et al. [21] introduced a four-facet framework (artifacts, producers, concepts, gatekeepers), and the gatekeeper facet’s network proximity was measured based on interlocking editorial board membership of 58 journals from the information science and library science category. In the field of information and library science, Baccini and Barabesi [15,16] applied network analysis techniques to determine which are the most central and which are the most peripheral journals in the interlocking editorship network. Finally, Baccini et al. [22] compared the interlocking authorship, the interlocking editorship, and the co-citation networks in statistics, economics, and information and library sciences.

The current studies investigating the interlocking editorship network are alike in that they almost exclusively focus on the journal level. We have, however, no information regarding the geographical patterns of the co-editor network. The main goal of this paper is to map the geography of the global co-editor network in the field of oncology; we analyze this latter research field using network techniques in this study due to the following reasons: Cancer is a leading cause of death worldwide [23], making it one of the main research areas globally. Whereas in the case of some fields, most of the global research activity is geographically tied to specific locations (e.g., particle physics requires big and expensive research infrastructure maintained by the richest nations and communities), and in the case of other research fields, a spatial bias is observed (e.g., in some countries, the social sciences do not belong to the mainstream research areas), cancer research is the focus in most countries. That is, there is a high likelihood of many researchers from multiple countries across the world participating in the work of oncology journals’ editorial boards, making the composition of such boards heterogeneous.

We investigated the geography of oncology journals’ co-editor network through the lens of cities. We involved 244 Web of Science–indexed oncology journals in the analysis that, in 2020, were edited by 13,342 scholars from 878 cities across the world occupying a total of 17,774 editorial board positions. By using network analysis techniques, we examined and mapped the interrelations between cities. We sought to answer the following research questions: 1) By which structure can the oncology journals’ co-editor network be described? 2) Which cities are the most interconnected in the network? 3) Can we characterize the network with a multipolar pattern?

## 2. Data and methodology

### 2.1 Data collection

The data collection of oncology journals’ editorial boards was conducted in 2020. Oncology journals were selected based on the research area classification of Web of Science (WoS), which, in the 2019 edition of the Journal Citation Reports, listed 244 journals under the heading “Oncology”. The affiliation data of editors were collected manually from the journals’ websites. When compiling the dataset, we excluded the inactive editorial board positions (e.g., honorary and founding editors) and the journals’ administrative staff (e.g., science writers and journal managers) [24] from the further analysis. The first draft of the dataset contained a total of 17,999 editorial board positions. To increase data accuracy, we checked each editor’s affiliation information one by one through the “Authors Search” tool of WoS. In many cases, we found that the editors neglected to update their affiliation information, or the journals indicated the affiliation information, even the name of the persons serving as editors, incorrectly. We also realized that some journals neglected to remove the name of deceased people from the editorial board (one of the researchers passed away in 2008). Besides, the affiliation information of some editors was impossible to detect.

After implementing all the necessary corrections and modifications, a total of 17,774 editorial board positions remained in the dataset. The 17,774 editorial board positions were occupied by a total of 13,342 scholars, which implies that many served as editor for more than one journal (see this issue thoroughly explained by Baccini and Barabesi [15,16]). In this study, we construct the co-editor network of oncology journals by considering the aggregate number of editorial board positions occupied by each scholar. That is, if a scholar serves as editor for five journals, they are technically an editor five times, and thus when constructing the network, their editorship must be counted five times. Consequently, the co-editor network refers to the network of editorial board positions (henceforward: editors) rather than that of scholars.

The oncology journals are edited by an average of 73 scholars. The editorial boards’ size varies from journal to journal; for example, Advances in Cancer Research is edited by only two persons, whereas the editorial board of BMC Cancer consists of 590 editors.

### 2.2 Geographical location of editors

The editors of the 244 journals are affiliated with 2,554 organizations being located in 1,329 settlements worldwide. These settlements are highly different in terms of size and population, and they occupy various hierarchical positions in the urban network. To increase consistency, we investigated the network connectivity of metropolitan areas rather than any type of settlement.

The delineation methodology of metropolitan areas to increase the comparability of the data was crucial. In spatial scientometrics, different methods are employed to merge localities into larger spatial units [25]. Some researchers developed case-specific delineation methods and created metropolitan areas exclusively for analytical purposes [26–31], whereas other researchers used already-existing metropolitan area classifications developed by national and international organizations [32–35]. In this study, we employed the latter approach and merged settlements according to the metropolitan area classification of the European Spatial Planning Observation Network (ESPON) for Europe, the Office of Management and Budget (OMB) for the United States, and the Organisation for Economic Co-operation and Development (OECD) for Australia, Japan, and Latin America.

After carefully reviewing the geographical location of the 1,329 settlements (i.e., those being reported by the editors as the home to the organizations they are affiliated with), we merged them into 878 metropolitan areas.

### 2.3 Network methods

To characterize the inter-related system of the global co-editor network in oncology, we created a network in which two editors *i* and *j* are connected if they co-edit a journal. Because the size of journal editorial boards varies on a large scale, we weight co-editor links with the formula $w_{ij}=\sum_{m}\frac{\delta_{i}^{m}\delta_{j}^{m}}{n_{m}-1}$, where *w*_*ij*_ stands for the strength of the tie between editors *i* and *j*, $\delta_{i}^{m}$ and $\delta_{j}^{m}$ are 1 if editor *i* and editor *j* co-edit journal *m* and zero otherwise and *n*_*m*_ is the number of editors in the journal’s editorial board. This method suggested by Newman [36] attributes weaker weight to those co-editor links that take place in large editorial boards.

We aggregate the individual co-editor links to the city level by

$$W_{PQ}=\sum w_{ij},\;i\in P\;and\;j\in Q,$$

where *W*_*PQ*_ is the edges weight between cities *P* and *Q* that host editors *i* and *j*.

To separate the densely connected core from the periphery of the global co-editor network in oncology, we apply the rich-core method [37]. The advantages of this method are that it can be applied on weighted networks, it needs no parameters and prior knowledge about the network. The rich-core method utilizes the notion that high-degree core nodes are usually connected with other high-degree core nodes. The method follows three steps:

1. we rank cities in decreasing order in terms of their strength (sum of their links’ weights, *S*_*P*_ = ∑_*Q*_ *W*_*PQ*_);
2. for each city, we calculate the share of those *S*^*+*^ network neighbors that have higher strength than the focal city;
3. we define the limit of core at that P* city for which the $S_{P*}^{+}>\;S_{P}^{+}$ for all *S*_*P*_ > *S*_P*_.

To further segment the core of the global co-editor network, we use the Louvain algorithm [38] that partitions nodes into communities by hierarchical clustering. This algorithm maximizes the density of the links within communities as compared to the density of links across communities, a measure called modularity in network science [39]. The modularity Q of the network’s partition can be written as

$$Q=\sum_{k=1}^{K}\left[\frac{L_{k}^{w}}{L}-{\left(\frac{L_{k}}{L}\right)}^{2}\right],$$

where *L* is the sum of edge-weights in the core of the co-editor network, *L*_*k*_ is the sum of edge weights of group *k*, and $L_{k}^{w}$ is the sum of edge-weights within group *k*.

Finally, to characterize communities, we calculate the average impact factor (AIF) of co-edited journals by resident editors in cities that are part of the community. AIF is the mean of impact factors of all journals that are co-edited between cities *P* and *Q*. This approach enables us to quantify the impact of the co-editor community in case cities *P* and *Q* are within the same community and also characterize co-editor relations across pairs of communities.

## 3. Results

### 3.1 City level co-editor network of oncology journals

The editors of the oncology journals are located in 878 cities worldwide. Most cities (385) are in Europe, followed by Asia (245) and Northern America (171), whereas the rest of the world (i.e., Africa, Australia, and Latin America) hosts 77 cities only. Considering the geographical distribution of the editors across cities, it turns out that most editors are located in Northern American cities (8,254). The European cities are home to 5,400 editors, and 3,466 editors are from Asian cities (Table 1). The average number of editors per city in Europe (14.03) and Asia (14.15) is almost equal, whereas the Northern American cities concentrate a much higher average number of editors (48.27). Being home to a total of 654 editors (4.90% of all), cities in Africa, Australia and Oceania, and Latin America play a marginal role in the co-editor network.

**Table 1:** Top-10 cities in terms of editors and scholars by continent.

	Asia				Europe			
	City	Country	Aggregate number of editors	Number of scholars serving as editors	City	Country	Aggregate number of editors	Number of scholars serving as editors
1	Tokyo	Japan	558	414	London	United Kingdom	312	229
2	Seoul	South Korea	342	264	Milan	Italy	226	157
3	Osaka	Japan	210	162	Paris	France	220	159
4	Beijing	China	174	126	Rome	Italy	164	128
5	Shanghai	China	160	120	Amsterdam	Netherlands	126	84
6	Hong Kong	China	124	80	Barcelona	Spain	126	84
7	Nagoya	Japan	104	81	Munich	Germany	112	87
8	Guangzhou	China	96	63	Heidelberg	Germany	107	73
9	Singapore	Singapore	92	71	Naples	Italy	90	59
10	Taipei	Taiwan	72	53	Vienna	Austria	89	61
Total (245 cities)			3,466	2,723	Total (385 cities)		5,400	4,115
Top 10 share			55.74%	52.66%	Top 10 share		29.11%	27.24%
	Northern America				Rest of the World			
	City	Country	Aggregate number of editors	Number of scholars serving as editors	City	Country	Aggregate number of editors	Number of scholars serving as editors
1	New York	USA	905	616	Melbourne	Australia	143	105
2	Washington	USA	725	512	Sydney	Australia	127	89
3	Boston	USA	651	479	Brisbane	Australia	48	40
4	Houston	USA	604	405	São Paulo	Brazil	41	39
5	Los Angeles	USA	385	277	Adelaide	Australia	36	29
6	Philadelphia	USA	337	245	Mexico City	Mexico	29	29
7	San Francisco	USA	289	216	Buenos Aires	Argentina	23	16
8	Chicago	USA	239	170	Perth	Australia	20	19
9	Detroit	USA	214	148	Auckland	New Zealand	17	12
10	Raleigh-Durham	USA	186	129	Cairo	Egypt	13	12
Total (171 cities)			8,254	5,971	Total (77 cities)		654	533
Top 10 share			54.94%	53.61%	Top 10 share		75.99%	73.17%

We consider a journal’s editorial board to be a network of interconnected co-editors. Most oncology journals maintain international editorial boards; that is, the editorial boards have members from at least two countries. Notably, previous works define journal editorial boards to be international if they contain scholars from at least five or even eight countries [40,41]. Furthermore, Calver et al. [42] use statistical hypothesis testing to quantify the internationality of journals. However, our study is more focused on the geographical than the bibliometric aspects of the co-editor network, which implies that an editorial board should be considered international if the editors are located in at least two countries.

Some journals, however, are edited by scholars from a single country. Even in the latter case, the editors are generally located in different cities. In the co-editor network, the cities are interconnected by the journal’s editorial boards. Each city contains at least one editor, but the facts that the top 15 cities concentrate 35 percent of the editors, and 30 percent of the cities host 90 percent of the editors, indicate that the network is highly centralized. Four journals are edited by a single editor; thus, those journals are not part of the network analysis.

### 3.2 Description of the co-editor network

**Fig 1:**
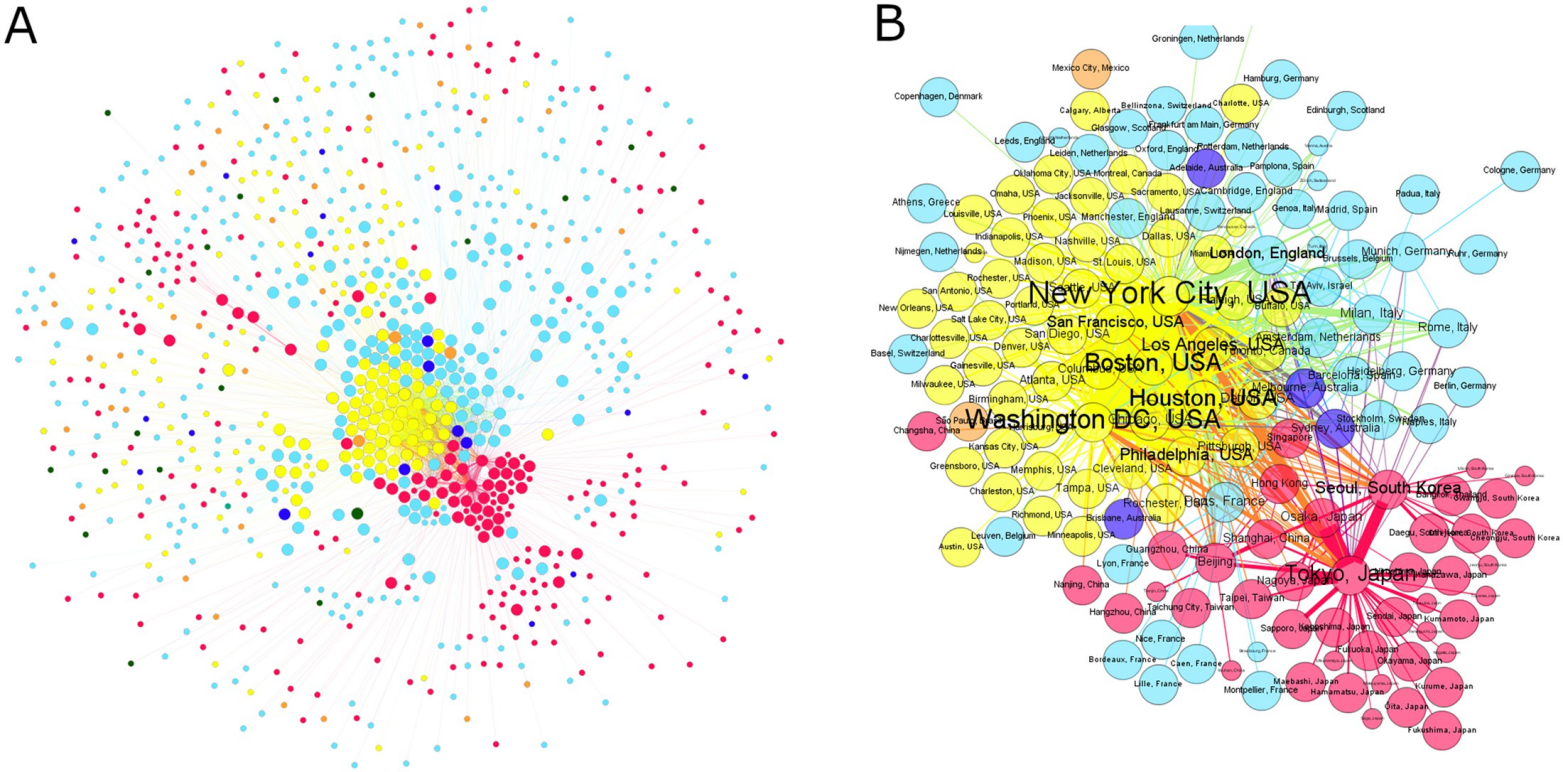
City-level co-editor network of oncology journals. **(A)** Visualization of the entire network. We keep all cities in the network by applying the maximum spanning method. Then, the strongest 10% of edges are added to this network to illustrate the density of the network core. Large nodes are part of the network core, and small nodes are in the network periphery (described in the Methods section). Nodes are colored by continents: Yellow—Northern America, red—Asia, light blue—Europe, dark blue—Australia and Oceania, orange—Latin America, black—Africa. **(B)** The asymmetric sub-core structure of the co-editor network. Node labels are proportional to the number of resident editors in cities. Edge thickness is proportional to the number of journals co-edited between the pairs of cities.

As can be seen in Fig 1A, the co-editor network is constituted by two main parts: a dense core containing 242 cities and a periphery of 636 cities that are loosely interconnected with the core cities (a brief description of the co-editor network’s periphery is located in S1 Material). The core cities contain 15,255 editors (85.83 percent), whereas the peripheral cities host 2,527 editors (14.17 percent). The core is characterized by an asymmetric dual sub-core structure (Fig 1B). The greater sub-core is formed by 74 Northern American cities, out of which 67 are in the United States. The Northern American sub-core is centered around New York, Washington, Boston, and Houston. These four cities account for 35 percent of the editors located in Northern American cities. Besides the Northern American sub-core, a well-outlined sub-core of Asian cities is established. Containing 49 cities, the Asian sub-core is smaller than the Northern American one. The focal point of the Asian sub-core is occupied by such Eastern Asian megacities as Tokyo, Seoul, Osaka, Beijing, and Shanghai. With 558 editors, Tokyo stands out from the cities located in the Asian sub-core. Based on the number of editors, Tokyo surpasses the second-ranked Seoul with 63 percent, whereas the difference between New York, the first-ranked Northern American city, and the second-ranked Washington is only 25 percent.

The 109 European core cities do not form such a well-delineated sub-core as the Northern American and the Asian cities but show a ringlike shape around the Northern American sub-core. The major European city in terms of editorship is London, followed by Milan, Paris, Rome, Amsterdam, Barcelona, Munich, and Heidelberg (Table 1). The proportion of the top-10 European cities in terms of editorship is less than 30 percent, whereas the proportion in the case of the top-10 Asian and Northern American cities is approximately 55 percent, respectively. This phenomenon can be explained by the higher number of European cities and the less concentrated and more homogeneous geographical distribution of the editors.

The 242 core cities establish a total of 2,120,601 co-editor links, out of which only 327,753 links (15.46 percent) are built with peripheral cities. The co-editor link distribution is characterized by the dominance of Northern America. The Northern American cities represent 30.58 percent of the core cities and host 51.69 percent of the editors affiliated with the core cities. In addition, 51.24 percent of the core cities’ co-editor links are built by the Northern American core cities (Table 2).

**Table 2:** Co-editor link distribution of core cities by continents.

	Number of editors	Number of core cities	Number of co-editor links built by the core cities	Share of co-editor links built by the core cities (%)	Share of co-editor links built among the core cities (%)	Share of co-editor links built with Asian cities (%)	Share of co-editor links built with European cities (%)	Share of co-editor links built with Northern American cities (%)	Share of co-editor links built with the rest of the cities (%)
Asia	2,765	49	436,216	20.57	80.17	41.87	25.01	29.81	3.31
*China*	*729*	*11*	*102*,*906*	*4*.*85*	*80*.*95*	*31*.*54*	*26*.*71*	*37*.*85*	*3*.*90*
*Japan*	*1*,*176*	*18*	*201*,*681*	*9*.*51*	*80*.*67*	*51*.*21*	*22*.*05*	*23*.*98*	*2*.*76*
*South Korea*	*439*	*7*	*72*,*606*	*3*.*42*	*77*.*39*	*41*.*59*	*26*.*44*	*28*.*65*	*3*.*97*
Europe	4,110	109	533,324	25.15	81.93	19.12	38.75	38.18	3.95
*Germany*	*739*	*22*	*97*,*648*	*4*.*60*	*81*.*50*	*17*.*47*	*44*.*83*	*34*.*37*	*3*.*33*
*France*	*409*	*11*	*43*,*944*	*2*.*07*	*84*.*51*	*15*.*17*	*38*.*46*	*42*.*54*	*3*.*83*
*Italy*	*733*	*15*	*108*,*467*	*5*.*11*	*79*.*54*	*23*.*91*	*37*.*00*	*35*.*14*	*3*.*95*
*United Kingdom*	*716*	*15*	*94*,*630*	*4*.*46*	*83*.*73*	*17*.*63*	*35*.*24*	*42*.*97*	*4*.*16*
Northern America	7,875	72	1,086,605	51.24	87.63	14.45	23.58	58.50	3.47
*Canada*	*317*	*7*	*39*,*694*	*1*.*87*	*86*.*44*	*14*.*09*	*27*.*53*	*54*.*01*	*4*.*38*
*United States*	*7*,*558*	*67*	*1*,*046*,*911*	*49*.*37*	*87*.*67*	*14*.*46*	*23*.*43*	*58*.*67*	*3*.*44*
Africa	13	1	1,263	0.06	78.70	19.08	35.63	41.81	3.48
Australia	391	6	46,430	2.19	84.58	19.10	31.02	45.67	4.21
Latin America	93	3	16,763	0.79	82.02	23.56	30.38	41.84	4.21

As Fig 1 demonstrates, the Northern American sub-core can be characterized by a highly dense structure. The cohesion between the Northern American (particularly the US) core cities is the strongest: 58.50 percent of the co-editor links of Northern American core cities are built among each other. This finding suggests that most editors located in Northern American cities are members of such journals’ editorial boards that dominantly or exclusively employ Northern American editors. This observation is also reinforced by the fact that in 111 journals’ editorial boards (i.e., in 45.49 percent of the oncology journals), the Northern American editors represent the majority (i.e., more than 50 percent of the editors are located in Northern American cities) (S2 Table).

Most Northern American co-editor links start from New York (118,635), Washington (90,740), Boston (83,962), Houston (83,895), Los Angeles (50,542), and Philadelphia (47,984), ensuring the central position of these cities in the Northern American sub-core. With 6,483 and 6,453 co-links, the New York‒Boston and New York‒Washington co-editor connection is the strongest in the world. In terms of co-editorship, the most significant international co-editor connection in the world exists between New York and Tokyo (2,258 co-links).

Only 23.58 percent of the Northern American co-editor links are built with European cities and 14.45 percent of them with Asian cities.

The ringlike pattern created by the European cities around the Northern American sub-core (Fig 1) is essentially due to two factors: 1) the strong cohesion between the Northern American cities (i.e., the high ratio of intracontinental links) and 2) the peculiar co-editor link distribution characterizing the European cities. As Table 2 shows, the proportion of links built among European cities and those built between European and Northern American cities is almost equal (38.75 vs. 38.18 percent); that is, the proportion of intracontinental co-editor links in the case of Northern America is much higher than for Europe. The proportion of links established by European cities with Asian cities only represents half of the intracontinental European links. These aforementioned facts explain why most European cities are centered around the Northern American sub-core and are less interconnected with the Asian sub-core.

Considering the composition of the journals’ editorial boards in terms of the editors’ origins, we can conclude that the European editors represent the majority in 62 journals’ editorial boards, which is only 55.86 percent of the value characterizing Northern America (S1 Table). Hence, for Northern American editors, more space is available to establish intracontinental co-editor links than for European editors.

The major European countries in terms of editorship are Germany, Italy, the United Kingdom (UK), and France. These four countries account for 57.80 percent of the number of European core cities, 63.19 percent of the number of editors, and 64.63 percent of the co-editor links established by European cities. In Table 2, some important differences can be detected in the co-editor link distribution of these countries. The editors affiliated with German cities build the highest ratio of co-links with editors located in European cities and construct the lowest ratio with Northern American editors. Although Germany produces the fifth-highest number of co-links in the network (97,648), 56.48 percent of the links belong to only eight journals, out of which seven are published by European publishing houses (BMC, Karger Publishers, and Spandidos Publications) or publish papers mostly in German (e.g., Strahlentherapie und Onkologie which itself contains 15,998 co-links).

As for France, it is one of the handful of countries that publish non-English language WoS-indexed oncology journals. These journals (i.e., Bulletin du Cancer, Cancer Radiotherapie, Psycho-Oncologie, and Oncologie) are platforms for mostly French-language cancer research publications and are dominantly edited by France-based scholars (more precisely: 87.78 percent of the editors are from France). However, as can be seen in Table 2, the ratio of the co-editor links between French core cities and Northern American cities is slightly higher than between French core cities and European cities.

The United Kingdom traditionally maintains strong ties in science with the United States (UKRI, 2020), as also reflected in the high ratio of UK−US co-editor links. The Italian core cities maintain almost equal ratios of co-editor links between European and Northern American cities. However, as compared to the major European countries in terms of editorship, the Italian core cities have the strongest co-editor links with Asian cities (i.e., Italian editors are frequently employed by journals, the editorial boards of which contain many Asian editors).

Most co-editor links are established by London (36,612), followed by Rome (24,007), Milan (22,275), Paris (22,480), and Naples (21,377). In terms of the number of co-editor links, the European cities lag the Northern American ones. Considering the number of editors and the number of co-editor links, London is the top-ranked European core city. London, however, occupies a less dominant position in Europe than New York in Northern America due to differences in such indicators as the number of editors (London: 312 vs. New York: 905) and co-editor links (London: 36,612 vs. New York: 118,635). Furthermore, whereas the co-editor links built between London and European cities, and London and Northern American cities account for 33.47 percent and 45.24 percent of all of London’s co-editor links, respectively; New York is more involved in constructing intracontinental co-editor connections: the proportion of New York’s co-editor links with Northern American cities is 56.45 percent. With 485 co-editor links, the London‒Milan co-editor connection is the strongest in Europe, but the magnitude of the London‒New York connection in terms of co-editor links is 3.78 times higher.

Although there are a couple of oncology journals being dominantly edited by European scholars (most of which also contain many Northern American-based editors), the European editors more often have positions in such journals’ editorial boards, of which the majority are occupied by Northern American scholars (even if the journals are published by European publishing houses in association with European professional organizations).

At first glance, the Asian sub-core is characterized by a highly integrated pattern. However, if we dig more deeply, it turns out that among the major countries of the region (i.e., China, India, Japan, and South Korea), only loose intracontinental connections exist. By being home to the most core cities (18 out of 49) and editors (1,367 out of 3,466) and by building the most co-editor links (201,681), Japan is undoubtedly the major actor in the co-editor network of Asia. The Asian sub-core is centered around Tokyo. The Japanese capital built 79,792 co-editor links, which is 18.29 percent of all links established by Asian core cities. Tokyo constructs strong ties with its national and regional peers (Osaka and Nagoya, and Seoul and Beijing). The number of Tokyo‒Osaka co-editor links (6,148) is the third-highest in the world, surpassed by only the New York‒Boston and New York‒Washington connections. In addition, with 3,437 co-editor links, the Tokyo‒Seoul city dyad produces the strongest international co-editor connection globally. 48.64 percent of Tokyo’s co-editor connections are built with Asian cities, a ratio that exceeds the Asian average (Table 2). Considering the co-editor link distribution of other Japanese core cities, we can conclude that the ratio of the intra-Asian and particularly the domestic connections are much higher than for Tokyo (the ratio of intracontinental co-editor links for Osaka is 50.01 percent, Fukuoka: 52.53 percent, Sapporo: 55.55 percent, and Nagoya: 60.71 percent). A significant proportion of the Japanese editors are employed by such English-language oncology journals that are published for Japanese professional organizations by the Japanese branches of international publishing houses. For example, the editorial board of Cancer Science, a journal published by Wiley for the Japanese Cancer Association, comprises 262 editors, of whom 82.06 percent is from Japan.

In South Korea, the co-editor network is more centralized than in Japan because Seoul, the major South Korean hub in terms of editorship, is not followed by such dominant second-tier hubs than in the case of Japan, Tokyo is followed by Osaka, and Nagoya. Seoul hosts 342 editors, whereas Daegu, the second-ranked South Korean city, is home to 28 editors only. For Seoul, the intracontinental co-editor link ratio is less than 40 percent, and this ratio is naturally slightly higher for second-tier South Korean cities (for example, for Daegu, the intracontinental co-editor link ratio is 48.96 percent).

Surprisingly, China is characterized by a less-developed domestic co-editorship network. Considering the co-editor link distribution of the Chinese core cities, it turns out that the ratio of the intra-Asian co-editor links (31.54 percent) is lower than those built with Northern American cities (37.85 percent). Hence, the Chinese editors more frequently occupy positions in such journals’ editorial boards (regardless of being published by US- or Chinese-based publishing houses) that employ many Northern American editors but a relatively lower number of non-Chinese Asian editors. For example, Cancer Biology & Medicine, the journal of the China Anti-cancer Association, employs 50 editors from China, 13 editors from other Asian countries, and 34 editors from Northern America (and an additional 22 ones from Europe). This quasi-balanced structure of the editorial board does not characterize the Japanese journals for which the editorial boards contain mostly Japanese editors.

The major Chinese hubs of editorship are Beijing and Shanghai, followed by Hong Kong and Guangzhou (Table 1). Most co-editor links are produced by Beijing and Shanghai (22,039 and 21,868). In China, the domestic co-editor connections are not as significant as in the case of Japan. By investigating Beijing’s co-editor network, we find that the Beijing−Shanghai connection results in 653 links, which is surpassed by not only the Beijing−New York connection (804 links) but also the Beijing−Washington (733) and Beijing−Houston (728 links) connections.

### 3.3 Communities in the co-editor network core

By conducting hierarchical clustering using the Louvain community detection algorithm [38], we group cities into communities. The cities belonging to the same community are densely connected by co-editor links, whereas inter-community co-editor links are sparse. Thus, the investigation of communities in the core of the co-editor network provides another perspective to understand the geography of the co-editor network.

The core cities can be classified into six major communities that are labeled by their largest city in terms of the number of editors (those communities containing only one city are excluded from further analysis; Table 3). Northern America hosts two communities. The New York City community, which encompasses 105 core cities (43.4% of the core cities), is the most populous. The Los Angeles community contains 32 core cities (S4 Table). The number of editors is the largest in these two communities, and both stretch across continents. The most diverse community in city location is the New York City community. Surprisingly, this city community encompasses more European core cities (48) than North American ones (42). In fact, the New York City community contains more European core cities than do the European communities combined (S4 Table).

**Table 3:** Communities of core cities.

Label	Three largest cities	Number of cities	AIF	Editors				
				All	Q1 journals	Q2 journals	Q3 journals	Q4 journals
Los Angeles community	Los Angeles, Philadelphia, San Francisco	32	5.29	4,017	1,183	1,215	1,050	569
London community	London, Munich, Heidelberg	23	3.90	1,151	309	306	261	275
New York City community	New York City, Washington DC, Boston	105	3.74	5,333	1,526	1,584	1,284	939
Milan community	Milan, Paris, Madrid	21	3.80	1,062	290	273	226	273
Tokyo community	Tokyo, Seoul, Osaka	51	3.46	3,438	784	1,170	722	762
Mumbai community	Mumbai, Delhi, Budapest	7	1.85	192	15	31	49	97

**Fig 2:**
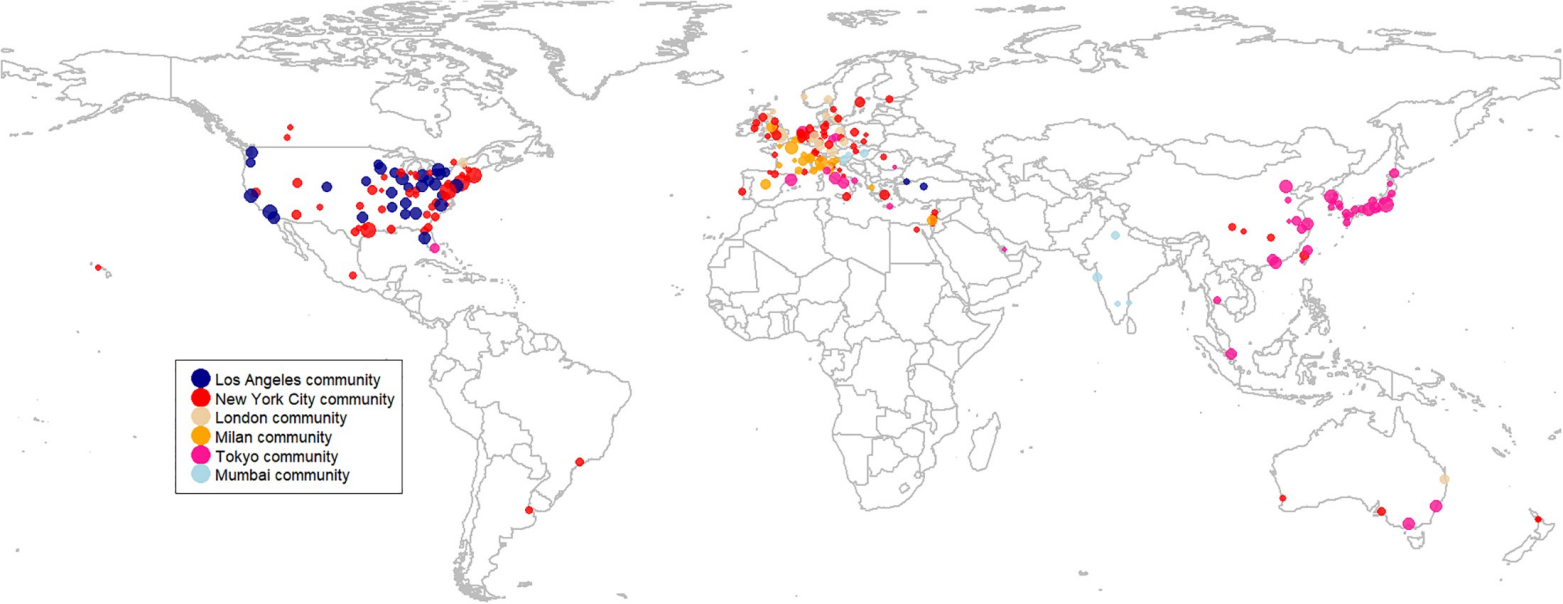
Mapping cities constituting the network by communities. Node colors represent the communities to which the cities belong. Node size is proportional to the number of editors resident in the city (log-transformed). Created by own data, with base map of Natural Earth licensed as CC BY.

The European communities are grouped into two communities centered on London and Milan. These two European communities are characterized by high homogeneity in the geography of cities because both the London and Milan communities almost exclusively constitute European core cities (Fig 2 and S4 Table).

We find two communities in Asia. The Tokyo community also contains several European cities but dominantly constitutes Asian core cities (71.7% of the Asian cities are located in the Tokyo community). Such major Chinese cities as Beijing, Guangzhou, Hong Kong, and Shanghai are also part of the Tokyo community. This is an astonishing finding because earlier we found that the Chinese cities constructed more intensive co-editor connections with Northern American cities than with their Asian peers. However, this pattern may change in the close future, and Chinese cities might become part of some US communities or establish their own community.

The smallest community in terms of the number of core cities is the Mumbai community, which includes four Indian and three Central and Eastern European cities.

Considering the number and geographical diversity of cities encompassed by a particular community, we can conclude that the New York City and Tokyo communities are the largest integrators for cities across the world. These mega-centers in the US and Asia integrate the gatekeeping function of editors residing on other continents as well. In contrast, the European communities of the co-editor network do not cross the continental borders. This is an important insight and signals the limited capacity of the European scientific system to control and monitor progress in other research areas.

**Fig 3:**
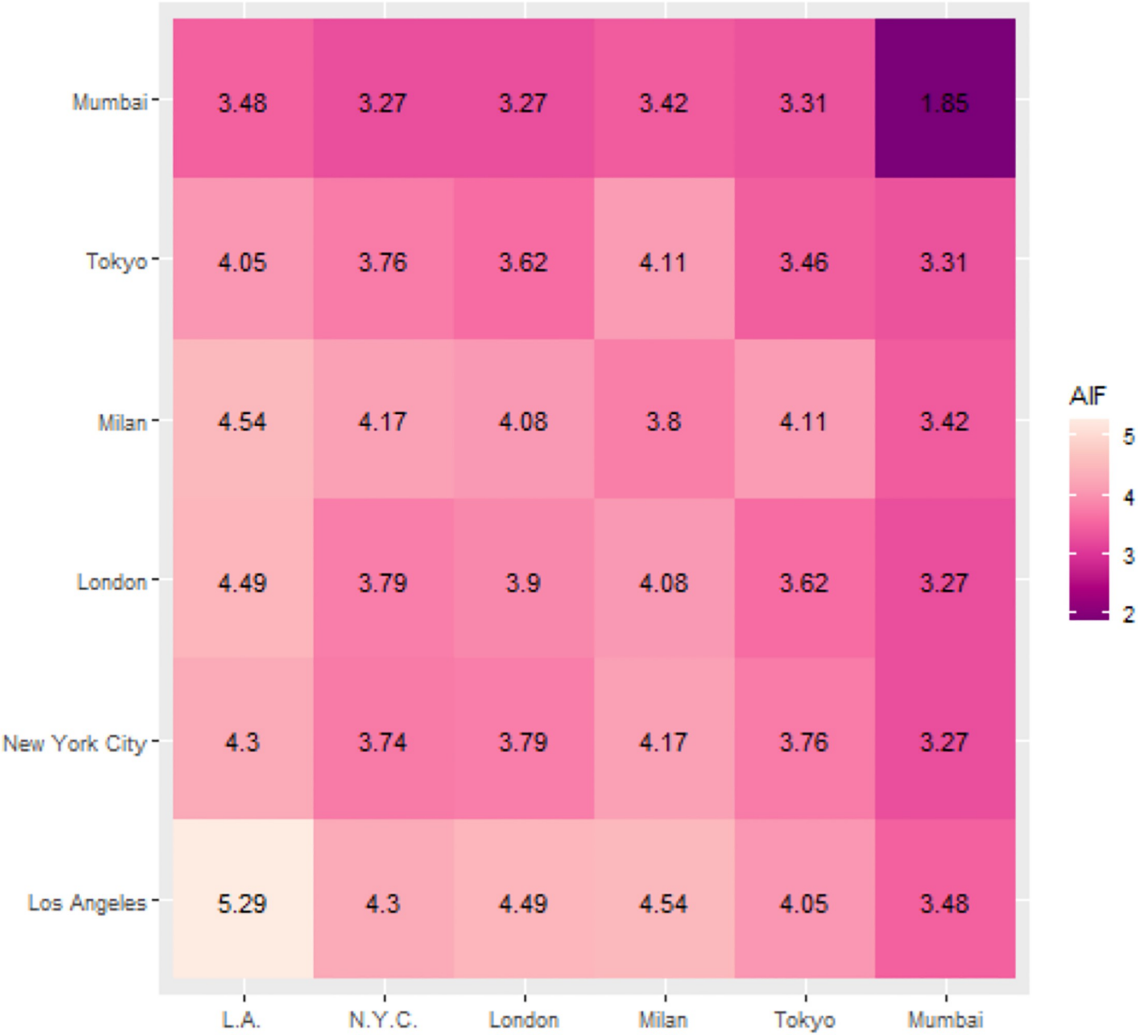
Average impact factor (AIF) of journals edited within and across city communities. Values in the diagonal represent the mean of journals’ impact factor co-edited across cities in the same community. Off-diagonal values represent the mean of journals’ impact factor co-edited across pairs of cities that are in the respective pairs of communities.

In the final step of the analysis, we characterize communities by the impact of the journals edited within them and in collaboration with editors in other communities (Fig 3). Editors located in the cities of the Los Angeles community are frequently employed by journals with a high impact factor (Table 3). Given the relatively small size of the Los Angeles community, this creates a homogenous group in which the journals edited within the community have an outstanding average impact factor (AIF). The New York City community and the two European communities have similar AIF values of journals co-edited within the community, whereas the Tokyo AIF score is somewhat lower. Editors from the Mumbai community occupy positions on the editorial boards of journals characterized by the lowest AIF.

An interesting observation in Fig 3 is that co-editor relationships across communities typically exist in higher-impact journals than co-editor relationships within communities, besides the Los Angeles community. For example, journals co-edited between the Milan and New York City communities have a higher AIF (4.17) than journals co-edited within the Milan (3.8) or New York City communities (3.74). Certainly, the concentration of high-impact journals has an impact on this pattern such that co-edited journals with the Los Angeles community have the highest AIF in almost all dyadic relations between communities. Conversely, journals co-edited with the Mumbai community have a relatively low impact.

## Discussion and conclusions

In this paper, we mapped the global co-editor network in oncology. Our main goals were to identify the major hubs of the network (i.e., those cities that hosted the most editors and were the most interconnected with other cities) to investigate whether the network could be characterized by a core-periphery structure and to examine whether the co-editor network showed a multipolar (tripolar) pattern.

We found that the co-editor network in oncology is constituted by two main parts: a core being occupied by 242 highly interconnected cities and a periphery containing 636 cities that are loosely interconnected with the core cities. Most of the peripheral cities are not connected with each other. The core of the network shows an asymmetric dual sub-core structure. The greater sub-core is constituted by Northern American cities with New York City, Washington DC, Boston, Houston, and Los Angeles in the center, whereas the smaller cub-core is formed by Asian cities and is centered on Tokyo, Seoul, Osaka, Beijing, and Shanghai. Although many European cities are located in the core, they do not produce a well-outlined sub-core but rather form a ringlike shape around the Northern American sub-core. Even the most dominant European core cities (i.e., London, Milan, Paris, Rome) contain a significantly smaller number of editors and construct a lower number of co-editor links than their Northern American and Asian peers. The weak positions of the European cities in the co-editor network are also demonstrated by the community detection analysis: whereas the major Northern American and Asian communities (i.e., the New York City and Tokyo communities) include many European cities, the two European communities—London and Milan—encompass only European cities (the New York City community contains more European cities than London and Milan combined).

To give a reasonable explanation of the characteristics of the co-editor network with a particular focus on the core, we must go down the journal level.

Most editors, more precisely, 44.1 percent of them, are located in the United States, followed by Japan, Italy, China, and Germany with ratios of 7.7, 5.7, 5.3, and 5.1 percent, respectively. In 111 oncology journals (45.5 percent of all), the Northern American editors represent the majority of the journal’s editorial board. In the case of 11 journals, the editorial boards are constituted by Northern American editors exclusively (S2 Table). There are 62 journals in which the European editors comprise more than 50 percent of the editorial board, and the Asian editors represent the majority in 22 journals’ editorial boards only. In addition, the Northern American editors account for 53.8 percent of all editors located in the most impactful journals’ editorial boards (i.e., in Q1 journals), whereas, for example, the ratio of the European editors is the highest in the less-impactful journals’ editorial boards (i.e., in Q4 journals) (S3 Table). In conclusion, the Northern American editors, particularly US-based ones, dominate the co-editor network in oncology.

The hegemony of the United States in global science is a well-known phenomenon. However, as Heinze et al. [43] point out, in the 2000s, Northern America’s hegemony began to be challenged by the European Union and Asia-Pacific scientific powers (e.g., China, Japan, and South Korea). Some other studies also corroborate this finding by demonstrating that the share of the United States in the global publication output and in the production of top-1 and top-10 percent highly cited papers has been gradually declining for decades [8,44,45].

However, we found that, despite its declining share in the global publication output, the United States still occupies the leading position in the control of global science. This finding reinforces the results of Braun and Dióspatonyi’s work published in 2005 [40], which demonstrated the role of the United States as the top gatekeeper nation of global science. Publishers across the world but outside of the United States tend to employ US-based scholars as editors because such an approach might help increase the journal’s prestige (see, for example, Hodgson and Rothman [46], and Paasi [47]). This strategy is frequently applied in China and some European countries. In the editorial board of journals published by Chinese publishing houses, the share of US-based editors is 35.6 percent, whereas that of the Chinese editors is 25.7 percent. Furthermore, the Chinese journals prefer to employ American editors rather than European ones. The Japanese journals, however, follow a different strategy: the Japanese editors account for 75.8 percent of the composition of the editorial boards. This strategy helps Tokyo occupy a central position in the Asian co-editor network.

We can conclude that the articulation of the global co-editor network in oncology is impacted by a sort of prestige effect. For many journals across the world, primarily for those published in China and Europe, this is a strategy to involve more American scholars in the journal’s editorial board to increase the journal’s prestige. For US-based journals, however, the additional value of employing non-American editors is uncertain. For example, the editorial board of CA-A Cancer Journal for Clinicians, the most impactful journal in 2020, is constituted by American editors exclusively. US-based oncology journals are more interested in employing American editors from the leading national cancer centers and universities, of which most are located in such metropolitan areas as New York, Boston, Houston, Los Angeles, and San Francisco. These metropolitan areas concentrate many top-ranked oncology institutions. The New York metropolitan area, for instance, is home to seven institutions involved in cancer research with more than 50 editors, respectively (including the Memorial Sloan Kettering Cancer Center, which provides 285 editors on its own); that is, besides the prestige effect, which favors editors from the United States, the geographical concentration of editors is supported by the size effect as well. These two effects combined help large US cities occupy the central position in the core of the co-editor network.

The limitation of this research is that we investigated the geographies of the co-editor network in the case of a single research field. Although we chose to analyze the spatial aspects of the co-editor network in oncology, a research field with wide geographical coverage, we assume that other research fields can be characterized by a different co-editor network pattern. In addition, it would be interesting to examine the temporal dynamics of co-editor networks; however, because data acquisition even back to some years is rather problematic, such an analysis would appear to be highly challenging.

### Software

The network methods and mapping were made with R (version number 4.0.5) and Gephi (version number 0.9.2).

## Supporting information

S1 Table Number and share of peripheral cities by continents. (PDF)

S2 Table Composition of editorial boards in terms of the share of editors from different continents. (PDF)

S3 Table Relationship between the share of editors from different continents and the Q-classification of journals as defined by WoS. (PDF)

S4 Table Classification of the core cities into communities. (PDF)

S1 Material Description of the periphery of oncology journals’ co-editor network. (PDF)
