# Supplementary material for: Geographies of the global co-editor network in oncology
Source: PLoS One. 2022 Mar 17;17(3):e0265652. doi: 10.1371/journal.pone.0265652 (PMC8929652; doi:10.1371/journal.pone.0265652)
Supplement: S1 Table — (PDF) [file pone.0265652.s001.pdf]

S1 Table. Number and share of peripheral cities by continents

	Africa	Asia	Australia	Europe	Latin America	Northern America
Number of peripheral cities	18	195	13	277	36	97
Share of peripheral cities to those located on the continent (%)	94.74	79.92	68.42	71.76	92.31	56.73
Share of peripheral cities to all peripheral cities (%)	2.83	30.66	2.04	43.55	5.66	15.25
