# Supplementary material for: Geographies of the global co-editor network in oncology
Source: PLoS One. 2022 Mar 17;17(3):e0265652. doi: 10.1371/journal.pone.0265652 (PMC8929652; doi:10.1371/journal.pone.0265652)
Supplement: S2 Table — (PDF) [file pone.0265652.s002.pdf]

S2 Table. Composition of editorial boards in terms of the share of editors from different continents.

Data show, for example, that 213 journals' editorial boards do not contain a single editor from Africa, and there are 11 journals of which the editorial board is constituted by Northern American editors exclusively.

	Number of journals of which the editorial board contains a particular share of editors from					
Share of editors in the journals' editorial boards	Africa	Asia	Australia	Europe	Latin America	Northern America
0%	213	46	100	18	170	15
0.01–25.00%	31	152	144	97	74	57
25.01–50.00%	0	24	0	67	0	61
50.01–75.00%	0	9	0	37	0	61
75.01–100%	0	11	0	15	0	39
100%	0	2	0	10	0	11
Total number of journals	244	244	244	244	244	244
