# Supplementary material for: Geographies of the global co-editor network in oncology
Source: PLoS One. 2022 Mar 17;17(3):e0265652. doi: 10.1371/journal.pone.0265652 (PMC8929652; doi:10.1371/journal.pone.0265652)
Supplement: S3 Table — (PDF) [file pone.0265652.s003.pdf]

S3 Table. Relationship between the share of editors from different continents and the Q-classification of journals as defined by WoS.

Based on the impact factor of journals being classified into a particular research area, WoS divides journals into four quarters. The Q1 class contains journals with the highest, and the Q4 class contains journals with the lowest impact factor.

Q rank	Share of editors from core cities (%)	Share of editors from peripheral cities (%)	Share of editors from Asian core cities (%)	Share of editors from European core cities (%)	Share of editors from Northern American core cities (%)	Share of editors from Asian peripheral cities (%)	Share of editors from European peripheral cities (%)	Share of editors from Northern America peripheral cities (%)
Q1	90.84	9.16	12.36	23.53	52.59	2.12	5.50	1.21
Q2	86.76	13.24	18.46	19.94	45.64	3.63	6.24	2.48
Q3	84.65	15.35	13.64	22.37	45.57	4.03	7.91	2.20
Q4	79.61	20.39	17.57	28.18	30.74	6.52	9.96	2.69
