# Supplementary material for: Geographies of the global co-editor network in oncology
Source: PLoS One. 2022 Mar 17;17(3):e0265652. doi: 10.1371/journal.pone.0265652 (PMC8929652; doi:10.1371/journal.pone.0265652)
Supplement: S4 Table — (PDF) [file pone.0265652.s004.pdf]

S4 Table. Classification of the core cities into communities

Data show that in terms of the location of core cities, the New York City community has global geographical coverage, making it the only global community.

	African cities	Asian cities	Australian cities	European cities	Latin American cities	Northern American cities
Los Angeles community		2				30
London community			1	21		1
New York City community	1	8	3	48	3	42
Milan community		1		20		
Tokyo community		38	2	10		1
Mumbai community		4		3		
