# Supplementary material for: Geographies of the global co-editor network in oncology
Source: PLoS One. 2022 Mar 17;17(3):e0265652. doi: 10.1371/journal.pone.0265652 (PMC8929652; doi:10.1371/journal.pone.0265652)
Supplement: S1 Material — (PDF) [file pone.0265652.s005.pdf]

S1 Material. Description of the periphery of oncology journals' co-editor network

Whereas 85.78 percent of the editors are located in the core cities, the periphery of the oncology journals' co-editor network encompasses 72.44 percent of the cities constituting the entire network. These facts suggest that a major difference exists between the average numbers of editors located in core vs. peripheral cities. The average number of editors per core city is 63.00, whereas the average number of editors per peripheral city is 3.97. Naturally, there are some core cities that host only a couple of editors. For example, in Ōtawara, a core city from Japan, as few as six editors are located. Nevertheless, 278 out of the 636 peripheral cities host only one editor, restricting those cities' capability to establish strong co-editor connections. Yet, the astonishing fact is that some peripheral cities do host a significant number of editors. More specifically, there are 51 peripheral cities across the world that are home to more than 10 editors, and with 89 editors, Vienna, the largest peripheral city in terms of editorship, is ranked 10th in the global ranking of cities (see Table 1).

If we observe the geographical distribution of the peripheral cities, an extraordinary pattern can be recognized. In the case of Africa and Latin America, the ratio of peripheral cities to all cities located in those continents is above 90 percent (Table 4); that is, most of those cities are barely integrated into the co-editorship network. This ratio is the lowest for Northern America, a factor that also contributes to the formation of the extremely dense Northern American sub-core. Almost three-quarters of the European cities are located on the periphery. In addition, with 277 cities (43.55 percent of all), Europe represents the largest group within the peripheral cities.
